# Concizumab for the management of heavy menstrual bleeding in a woman with mild hemophilia A

**DOI:** 10.1016/j.rpth.2026.106979

**Published:** 2026-09-24

**Authors:** Yoko Mizoguchi, Maiko Shimomura, Yuko Teraoka, Teruhisa Fujii, Satoshi Okada

**Affiliations:** 1 Department of Pediatrics, Hiroshima University Graduate School of Biomedical and Health Sciences, Hiroshima, Japan; 2 Department of Obstetrics and Gynecology, Hiroshima University Hospital, Hiroshima, Japan; 3 Division of Transfusion Medicine, Hemophilia Treatment Center, Hiroshima University Hospital, Hiroshima, Japan

**Keywords:** concizumab, hemophilia A, menorrhagia, tissue factor pathway inhibitor

## Abstract

**Background:**

Heavy menstrual bleeding (HMB) is a common bleeding manifestation in women with hemophilia A and may impair quality of life.

**Key Clinical Question:**

Can concizumab control refractory HMB in a woman with mild hemophilia A who cannot tolerate conventional therapies?

**Clinical Approach:**

An 18-year-old woman with mild hemophilia A had prolonged menstruation. Combined oral contraceptives improved bleeding but were discontinued because of mood-related adverse effects. Extended half-life factor (F)VIII concentrates shortened menstruation but caused significant burden due to repeated venipuncture. Following shared decision making, subcutaneous concizumab was initiated. During concizumab treatment, menstrual duration was generally maintained at 7 to 11 days per cycle. Menstrual control deteriorated after temporary concizumab dose reduction and remained inadequate despite the addition of tranexamic acid, prompting restoration of the previous concizumab dose. Subsequent concizumab concentration and D-dimer levels were within their respective target or reference ranges.

**Conclusions:**

Concizumab provided menstrual bleeding control broadly comparable with intravenous FVIII replacement while reducing treatment burden. This single case does not establish concizumab as a treatment for HMB in women with mild hemophilia A, but its potential role in carefully selected patients warrants further investigation.

## Essentials

• Heavy menstrual bleeding can greatly affect women with mild hemophilia A.
• The pill was not tolerated; factor VIII worked, but intravenous injections caused stress.
• Subcutaneous concizumab maintained menstrual bleeding control for 14 months.
• Concizumab may reduce treatment burden, but its wider use needs further study.

## Graphical abstract

**Figure:**
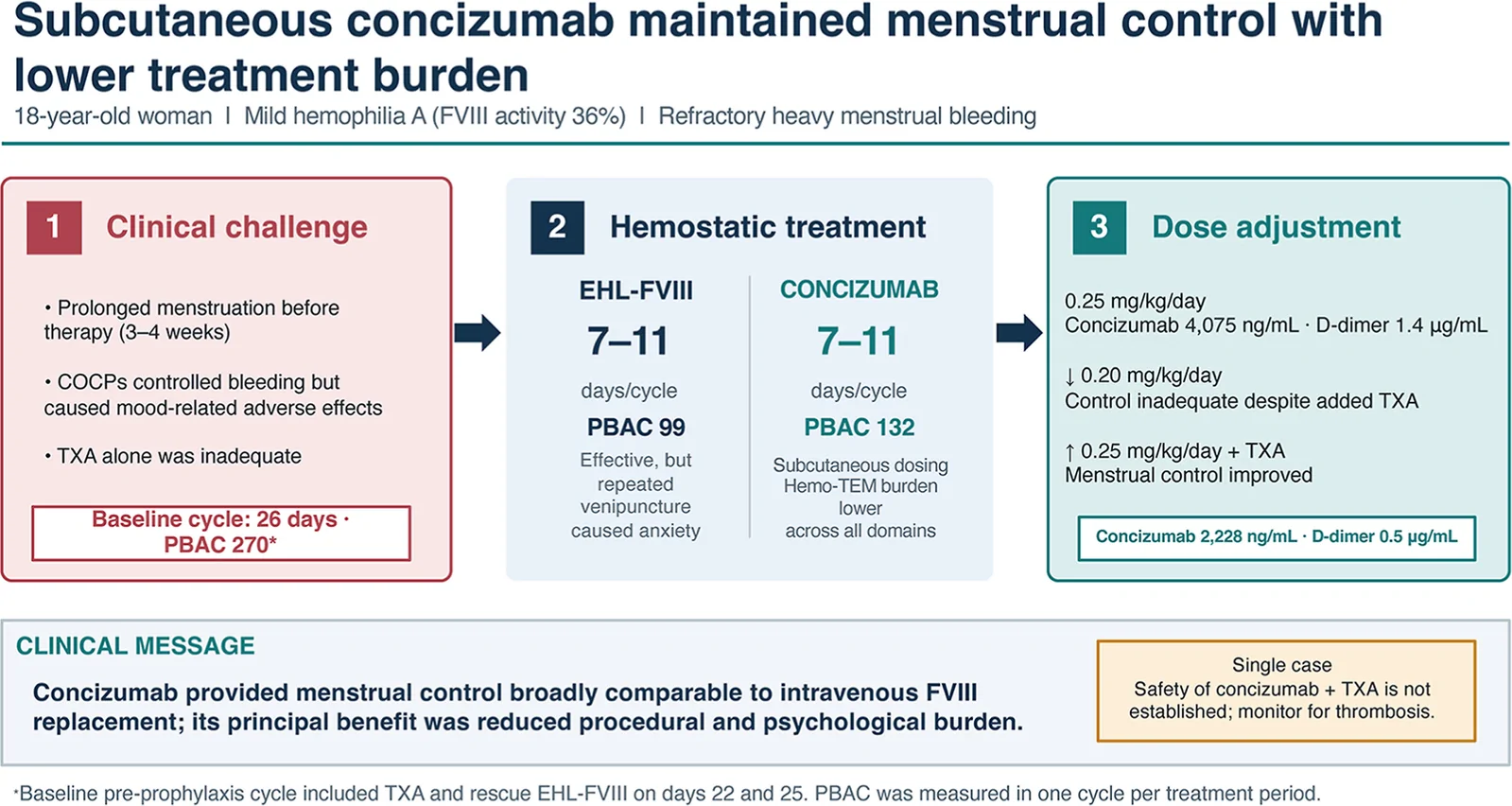

## 1 Introduction

Hemophilia A is an X-linked recessive bleeding disorder caused by deficiency of coagulation factor VIII. Approximately one-third of women and girls who carry a pathogenic *F8* variant experience clinically significant bleeding symptoms, including heavy menstrual bleeding (HMB) [[1], [2], [3], [4]]. HMB is particularly common and can severely affect quality of life (QOL); however, patients and clinicians often underestimate its severity [5,6]. The Pictorial Blood Assessment Chart (PBAC) provides a semiobjective method for quantifying menstrual blood loss, facilitating diagnosis and assessment of treatment efficacy [7].

Management strategies for HMB in women with hemophilia A include gynecologic interventions, such as levonorgestrel-releasing intrauterine systems (LNG-IUS) and combined oral contraceptives (COCPs), which aim to reduce endometrial proliferation and menstrual blood loss [8,9]. Hematological approaches encompass tranexamic acid (TXA), desmopressin, and factor replacement therapy [10]. While intranasal desmopressin is available in many countries, it is not approved in Japan, leaving patients dependent on intravenous factor replacement when hemostatic therapy is required. Novel subcutaneous therapies, such as concizumab—a humanized anti–tissue factor pathway inhibitor monoclonal antibody—may provide practical advantages by enabling effective hemostatic control while minimizing procedural burden [[11], [12], [13]].

## 2 Case

The patient was an 18-year-old woman with mild hemophilia A whose father had severe hemophilia A. Her body mass index was 22.3 kg/m^2^, indicating a normal nutritional status. At 12 years of age, she experienced her first menstruation, which lasted for approximately 1.5 months. During the subsequent untreated period, before initiation of any hormonal or hemostatic therapy, she recalled that her menstrual periods generally lasted for ∼3 to 4 weeks and were interspersed with amenorrheic intervals of 1 to 7 days. She subsequently visited the department of Obstetrics and Gynecology, where gynecologic imaging studies revealed no abnormalities. Initial hormonal therapies, including cyclic estrogen–progestin therapy and progestin preparations, did not adequately control the bleeding. Management with COCPs reduced menstruation to ∼4 days per cycle; however, the patient developed emotional instability as a side effect and strongly refused to continue hormonal therapy. At 18 years of age, she was referred to our department, while receiving COCPs, with adequate menstrual control and no evidence of iron-deficiency anemia. At the initial evaluation, the hemoglobin concentration was 13.5 g/dL, and the serum ferritin level was 15.5 ng/mL. Ferritin was not measured subsequently because the patient did not develop anemia during follow-up. She had no history of joint bleeding or uncontrollable mucosal bleeding other than HMB. Previous laboratory evaluation performed at another institution at 15 years of age demonstrated an activated partial thromboplastin time of 35.9 seconds, FVIII activity of 38% measured using a 1-stage clotting assay, and von Willebrand factor activity of 80%. The patient had blood type A. At presentation to our hospital, while the patient was receiving COCP therapy, activated partial thromboplastin time was 36.1 seconds and FVIII activity was 36% by 1-stage clotting assay. Subsequent FVIII activity measurements, including those shown in Figure 1, were performed using the chromogenic substrate assay, which was routinely used at our institution. The baseline FVIII activity measured by chromogenic substrate assay while the patient was not receiving FVIII replacement therapy was 33.8% (Figure 1). The persistently reduced FVIII activity in the presence of normal von Willebrand factor activity was consistent with a diagnosis of mild hemophilia A. After referral, the patient prospectively recorded menstrual bleeding on a daily basis using a structured pictorial diary. After discontinuation of COCPs, menstrual bleeding persisted despite TXA administration, corresponding to a PBAC score of 270. Extended half-life (EHL)-FVIII efraloctocog alfa was administered as rescue treatment on days 22 and 25, and bleeding ceased on day 26. This cycle was designated as the baseline preprophylaxis cycle (Figures 1 and 2A, B). Because bleeding cessation followed rescue EHL-FVIII administration, the recorded duration of 26 days should not be interpreted as the natural duration of a cycle treated with TXA alone. During subsequent menstrual cycles, several adjustments were made to the dose and timing of EHL-FVIII administration to establish an effective and feasible regimen. Ultimately, intravenous administration of efraloctocog alfa at 40 to 60 IU/kg every 3 days for a total of 3 doses, beginning on the first day of menstruation, achieved reproducible menstrual control and shortened menstrual duration to 7 to 11 days. A PBAC score of 99 was prospectively recorded during 1 menstrual cycle under this established EHL-FVIII regimen (Figure 1 and 2A, B). Despite only mildly reduced FVIII activity, the reproducible response to FVIII replacement supported impaired hemostasis as a major contributor to her prolonged menstrual bleeding. However, repeated venipuncture induced significant anxiety and procedural burden, negatively affecting adherence and overall QOL. Finally, the patient expressed reluctance to continue intravenous therapy. Alternative treatment options, including LNG-IUS, reintroduction of COCPs, emicizumab, concizumab, and efanesoctocog alfa, were discussed; however, she declined these approaches except for concizumab. The reasons she chose concizumab included the use of a fine needle, the absence of a need for venous access, and the lack of a reconstitution procedure before administration. The patient declined LNG-IUS because of concerns regarding insertion procedures and a strong preference to avoid gynecologic interventions. Neither the patient nor her family members have any history of venous or arterial thromboembolism or cardiovascular diseases. The treatment plan was discussed in a multidisciplinary setting involving hematologists and gynecologists. Because concizumab was prescribed as part of routine clinical care and not within a research protocol, institutional review board approval was not required under local regulations. Written informed consent for publication of this case report was obtained from the patient. Following thorough counseling regarding the risk of thrombosis and the need for effective contraception as part of a shared decision making (SDM) process, the patient elected to initiate subcutaneous concizumab therapy. She agreed to discontinue concizumab immediately and contact both her hematologist and gynecologist if pregnancy was suspected or confirmed. Protein C, protein S, and antithrombin levels were normal. Following an initial dose of 1 mg/kg, subcutaneous injections of 0.2 mg/kg once daily were initiated. The target concentration range of concizumab was 200 to 4000 ng/mL. On day 30 of treatment, the plasma concentration of concizumab was low at 102 ng/mL, and the D-dimer level was <0.5 μg/mL (reference range, 0-0.5 μg/mL); menstrual control remained inadequate. Dose escalation of concizumab up to 0.25 mg/kg increased the plasma concentration to 1368 ng/mL (D-dimer: 0.7 μg/mL), resulting in stabilization of menstrual cycles to 7 to 11 days occurring approximately once per month, corresponding to a PBAC score of 132 (Figures 1 and 2A, B). Six months after initiation, routine laboratory evaluation revealed an elevated D-dimer level (1.4 μg/mL) exceeding the reference range, although the patient remained asymptomatic. At that time, plasma concentration of concizumab was 4075 ng/mL. Consequently, the dose of concizumab was reduced to the standard dose. However, following dose reduction, the menstrual period became longer in duration, despite no apparent increase in menstrual blood volume. After further counseling regarding thrombotic risk, TXA was added; however, adequate menstrual control was not achieved with the combination of TXA and concizumab at 0.20 mg/kg once daily. The concizumab dose was therefore restored to 0.25 mg/kg once daily while TXA was continued. After dose restoration, menstrual control improved, and the plasma concizumab concentration was 2228 ng/mL, with a D-dimer level of 0.5 μg/mL; both the values were within their respective target or reference ranges. No clinically apparent thrombotic events occurred. The treatment burden associated with both EHL-FVIII and concizumab therapy was evaluated using the Hemophilia Treatment Experience Measure (Hemo-TEM), which the patient completed at each treatment phase [14]. The Hemo-TEM is a validated tool for assessing treatment experience in persons with hemophilia, with scores ranging from 0 to 100, where higher scores indicate worse treatment burden. The patient completed the questionnaire at the end of the EHL-FVIII treatment period and after stabilization of the concizumab dosing regimen. Although Hemo-TEM is validated in people with hemophilia, it has not been specifically validated in women with hemophilia or in patients with HMB; therefore, it was used to capture the patient’s perception of treatment burden. The results demonstrated marked improvements across all parameters, indicating alleviation of both physical and psychological burdens with concizumab therapy (Figure 3). The main advantages were its minimal injection pain and quick administration. After 1 year and 2 months of the initiation of concizumab treatment, the patient experienced no adverse events, including thrombosis, allergic reactions, or injection-site complications, and reported an improved QOL owing to the reduced procedural burden.

**Figure 1:**
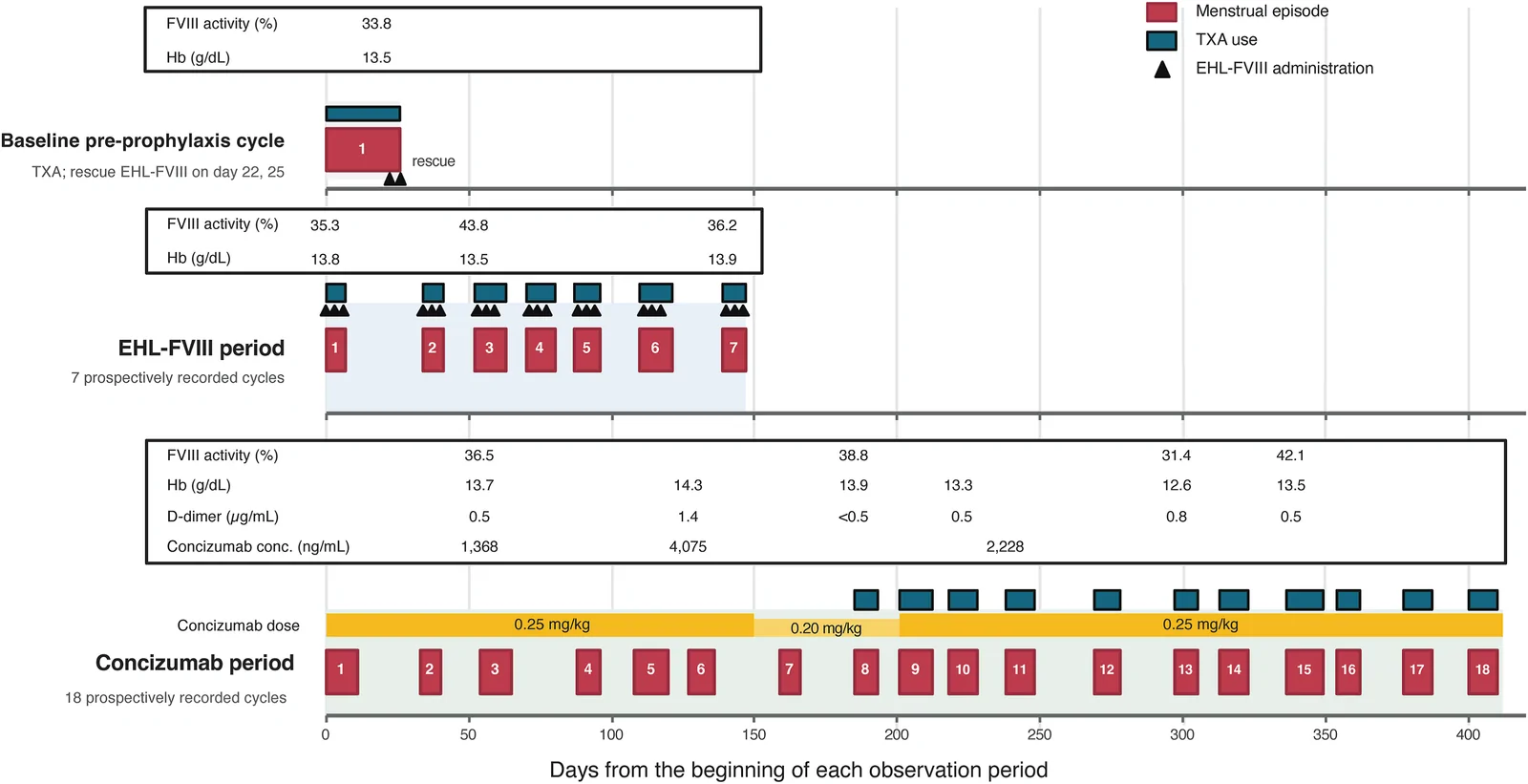
Menstrual episodes, treatment exposure, and laboratory findings during the 3 observation periods. Menstrual episodes during the baseline preprophylaxis cycle, extended half-life (EHL) factor (F)VIII treatment, and concizumab treatment are shown on relative time axes. Because the observation periods were nonconsecutive, the time axis was reset to day 0 at the beginning of each period. Each red bar represents 1 prospectively recorded menstrual episode; its position and width indicate the relative onset and duration, respectively. Numbers within the bars indicate menstrual cycle numbers. Blue rectangles indicate tranexamic acid (TXA) use, and black triangles indicate EHL-FVIII administration. The baseline preprophylaxis cycle occurred after discontinuation of combined oral contraceptives and before establishment of regular EHL-FVIII treatment. Despite TXA administration, menstrual bleeding persisted for 26 days and ceased after rescue EHL-FVIII administration on days 22 and 25. During subsequent menstrual cycles, the EHL-FVIII regimen was adjusted according to the clinical response. Once established, efraloctocog alfa at 40-60 IU/kg was administered on menstrual days 1, 4, and 7. The yellow bar indicates the concizumab dose. Available measurements of FVIII activity using the chromogenic substrate assay, hemoglobin, D-dimer, and concizumab concentration are shown at their corresponding relative time points. Exact calendar dates were omitted to preserve patient confidentiality.

**Figure 2:**
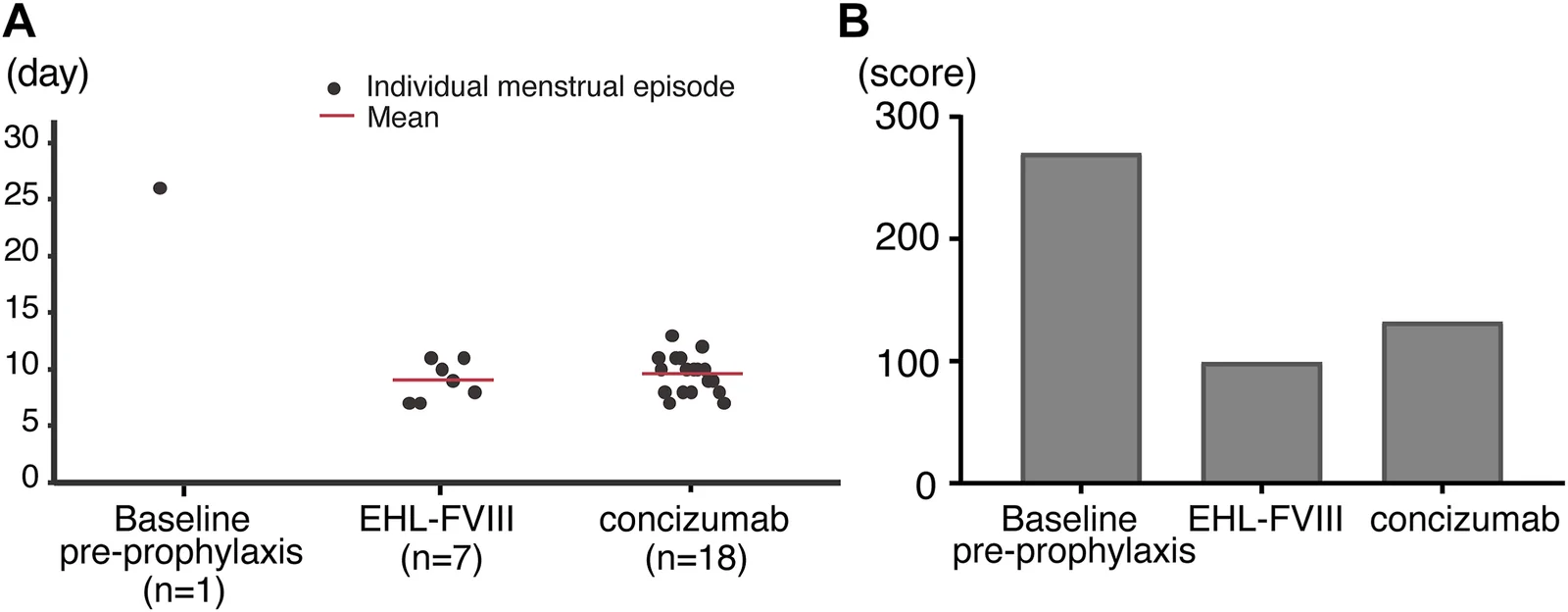
Menstrual duration and pictorial blood assessment chart (PBAC) scores during each treatment period. (A) Duration of individual menstrual episodes during the baseline preprophylaxis cycle, extended half-life (EHL) factor (F)VIII treatment, and concizumab treatment. Each dot represents 1 prospectively recorded menstrual episode. The numbers of evaluated episodes were 1, 7, and 18, respectively. Horizontal red lines indicate the mean menstrual duration during the EHL-FVIII and concizumab periods. No mean is shown for the baseline period because only 1 menstrual episode was evaluated. During the baseline cycle, menstrual bleeding persisted for 26 days despite TXA administration and ceased after rescue EHL-FVIII administration on days 22 and 25. (B) PBAC scores prospectively assessed during 1 menstrual cycle in each treatment period. The PBAC value for the baseline cycle reflects the cycle in which rescue EHL-FVIII was administered. Each PBAC value represents an individual-cycle measurement and not the mean of multiple cycles. The observation periods were nonconsecutive.

**Figure 3:**
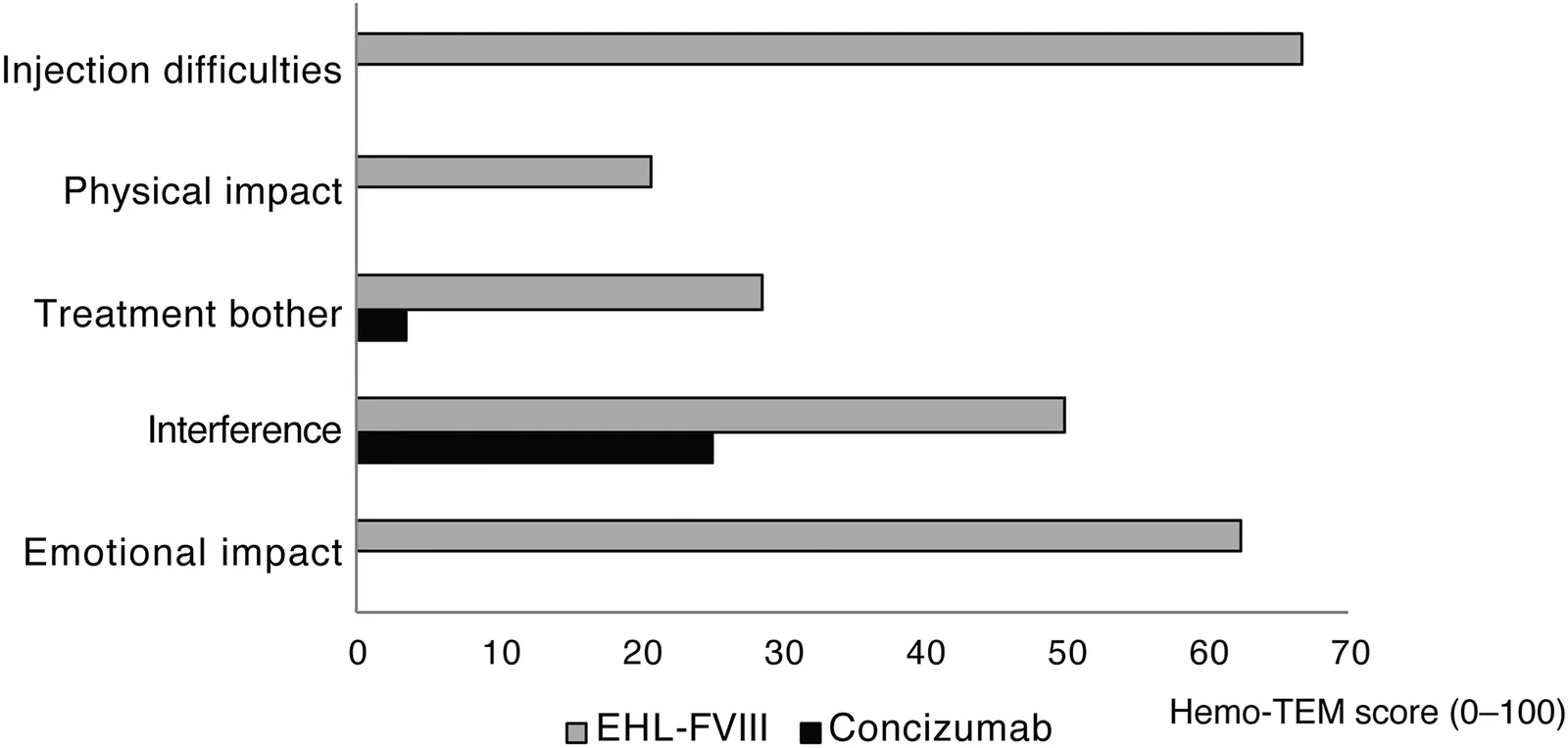
Patient-reported treatment burden during extended half-life (EHL) factor (F)VIII and concizumab treatment. Treatment burden was assessed using the Hemophilia Treatment Experience Measure (Hemo-TEM). The patient completed the questionnaire at the end of the EHL-FVIII treatment period and after stabilization of the concizumab dosing regimen. Domain scores are shown for injection difficulties, physical impact, treatment bother, interference with daily life, and emotional impact. Scores range from 0 to 100, with higher scores indicating greater treatment burden or a worse treatment experience. Gray bars represent scores during EHL-FVIII treatment, and black bars represent scores during concizumab treatment. Scores of 0 are represented by the absence of a visible bar.

## 3 Discussion

This case suggests the potential feasibility of concizumab for HMB in a woman with mild hemophilia A. Its hemostatic effect was broadly comparable, but not superior, to that of intravenous FVIII replacement therapy: menstrual duration remained 7 to 11 days, whereas the PBAC score was numerically higher with concizumab (132 vs 99). The main benefit was reduced treatment burden and better tolerability rather than improved bleeding control. Menstrual control remained inadequate after the addition of TXA at the reduced concizumab dose and improved after the concizumab dose was restored. However, because TXA was continued after dose restoration, the respective contributions of TXA and concizumab dose restoration cannot be separated in this single case. Although concomitant use of TXA and concizumab may theoretically increase thrombotic risk, no thrombotic complications were observed in our patient during 14 months of follow-up. Nonetheless, the absence of thrombotic events in this single case does not prove the safety of combining concizumab and TXA. Because clinical evidence regarding the safety of this combination is extremely limited, rigorous clinical monitoring for signs of thrombosis remains essential when these agents are used concomitantly.

Menstrual bleeding in hemophilia carriers can be prolonged and debilitating, impacting physical, emotional, and social well-being. While traditional factor replacement therapy effectively reduces bleeding, intravenous administration requires repeated venous access, which can be painful, anxiety inducing, and logistically challenging, particularly for adolescents and young adults. Previous studies comparing preventive goals between physicians and persons or caregivers with hemophilia have revealed differences in treatment preferences and values [15]. Physicians tended to prioritize clinical management and reduction of bleeding episodes, listing “zero bleeding per year” as the top priority. In contrast, patients and caregivers placed greater emphasis on QOL and treatment burden, prioritizing the ability to lead a normal daily life without being constantly aware of hemophilia. These findings suggest that, when determining optimal treatment strategies, it is essential to address patients’ and caregivers’ individual concerns and expectations and to incorporate SDM in the management process. From the perspective of treatment burden, subcutaneous therapy may be particularly advantageous for patients who are intolerant to hormonal therapy or who experience significant stress with repeated intravenous infusions. In our case, the introduction of concizumab through SDM enabled both effective bleeding control and a marked improvement in QOL.

Several limitations should be acknowledged. First, only 1 baseline preprophylaxis menstrual cycle was prospectively recorded, and bleeding cessation during this cycle followed rescue EHL-FVIII administration; therefore, the recorded duration does not represent the natural course under TXA alone. Second, PBAC was assessed during only 1 menstrual cycle in each observation period and therefore could not characterize within-period variability in menstrual blood loss. Although the patient did not develop anemia during follow-up, ferritin was measured only at the initial evaluation; consequently, longitudinal changes in iron stores could not be assessed. Third, the observation periods were nonconsecutive. In addition, TXA was added after concizumab dose reduction and was continued during subsequent dose restoration. Although adequate menstrual control was not achieved with TXA and the reduced concizumab dose, the respective contributions of TXA and concizumab dose restoration to the subsequent improvement cannot be determined from this single case.

In conclusion, concizumab provided menstrual bleeding control broadly comparable, but not superior, with that achieved with intravenous FVIII replacement in this woman with mild hemophilia A. Its principal benefit was the reduced physical and psychological treatment burden associated with subcutaneous administration, particularly given her difficulty with venous access and intolerance to hormonal therapy. This single case does not establish concizumab as a treatment for HMB in women with mild hemophilia A but suggests that its potential role in selected patients warrants further investigation.
